# Synthetic data generation by diffusion models

**DOI:** 10.1093/nsr/nwae276

**Published:** 2024-08-24

**Authors:** Jun Zhu

**Affiliations:** Department of Computer Science and Technology, BNRist Center, Tsinghua-Bosch Joint ML Center, Tsinghua University; Pazhou Laboratory (Huangpu), China

## Abstract

This paper provides a brief overview on diffusion models, which are extremely powerful on generating high-dimensional data, including images, 3D content, and videos, and provides insights for future work.

Sufficient and high-quality data are a prerequisite for building complex machine learning systems, especially those with a large number of parameters (e.g., ChatGPT). However, it is typically challenging or even impossible to acquire a sufficient amount of real data to train such systems. For example, auto-driving systems may need to learn from various accidental events in order to be reliable in driving, while collecting such real data is difficult or ethically infeasible. Unlike real data, synthetic data are artificially generated using algorithms without real-world occurrences. There are at least three advantages of synthetic data compared to real data: (1) synthetic data can be more *cost-effective*, as some real data (e.g. real vehicle crash data for auto-driving) can be extremely expensive; (2) synthetic data can be more *time-effective* to generate as they are not captured from real-world events and (3) synthetic data can be *privacy-preserving* as they only resemble real data, often with little traceable information about the actual data.

Given these advantages, it is becoming increasingly important to generate synthetic data for at least two purposes: (1) as complementary data to improve machine learning models, especially when real data are scarce or expensive to generate [1]; (2) as a great aid to human artists in computer-generated arts, e.g., by generating initial sketches, suggesting diverse artistic styles or even co-creating artworks [2]. To generate synthetic data in high dimensions, deep generative models (DGMs) are the most powerful approaches. Among various representative models like generative adversarial networks (GANs) [3], diffusion models [4,5] are the most widely used methods for modeling the distribution of continuous-domain data and generating new samples, because of their training stability and strong model capacity. Diffusion models can not only generate creative artist-styled images [6,7] by training with a large number of data examples, but also have the ability to generate novel samples when very few training samples are available [8].

Figure 1 illustrates the basic idea of a diffusion model, which consists of a forward diffusion process $\lbrace {q}_t\rbrace _{t\in [0,1]}$ that gradually adds Gaussian noise to a clean data point (e.g. an image) $\boldsymbol {x}_0 \sim q_0(\boldsymbol {x}_0)$, and then learns a reverse diffusion process $\lbrace p_t\rbrace _{t\in [0,1]}$ that gradually removes noise to finally generate high-quality data. The forward process is commonly defined as Gaussian distributions $q_{t}(\boldsymbol {x}_t|\boldsymbol {x}_0) := \mathcal {N}(\alpha _t\boldsymbol {x}_0, \sigma _t^2 \boldsymbol {I})$, where the hyperparameters $\alpha _t\ {\rm and}\ \sigma _t$ typically satisfy $\alpha _0\approx 1, \sigma _0\approx 0, \alpha _1\approx 0, \sigma _1\approx 1$. Moreover, let $q_t(\boldsymbol {x}_t) := \int q_t(\boldsymbol {x}_t|\boldsymbol {x}_0)q_0(\boldsymbol {x}_0)\mathrm{d}\boldsymbol {x}_0$ denote the marginal distribution at time *t*. Then, we can have $q_1(\boldsymbol {x}_1)\approx \mathcal {N}(\boldsymbol {0},\boldsymbol {I})$. In other words, the forward process constructs a trajectory from the data distribution to the (approximated) standard Gaussian distribution, and we only need to reverse such a process to draw samples from the data distribution. Concretely, the reverse diffusion process exists and is often defined by gradually denoising from the standard Gaussian $p_1(\boldsymbol {x}_1):= \mathcal {N}(\boldsymbol {0},\boldsymbol {I})$ with a *noise prediction network*  $\boldsymbol {\epsilon }_\theta (\boldsymbol {x}_t, t)$ that predicts the noise added to clean data $\boldsymbol {x}_0$. The unknown parameters of the network are learned by minimizing the objective


(1) \begin{eqnarray*}
&&\!\!\!\mathcal {L}_{\text{Diff}}(\phi) := \\
&&\!\!\!\mathbb {E}_{\boldsymbol {x}_0\sim q_0(\boldsymbol {x}_0),t\sim \mathcal {U}(0,1),\boldsymbol {\epsilon }\sim \mathcal {N}(\boldsymbol {0},\boldsymbol {I})} [\omega (t)\\
&&\ \ \ \ \ \ \ \ \Vert \boldsymbol {\epsilon }_\phi (\alpha _t\boldsymbol {x}_0+\sigma _t\boldsymbol {\epsilon }) - \boldsymbol {\epsilon }\Vert _2^2\big],
\end{eqnarray*}

where $\omega (t)$ is a weighting function that depends on time. Note that the variance of the denoising process has an analytical form and can be estimated in a training-free manner [9]. After training, we have approximately equal marginal distributions (i.e. $p_t\approx q_t$) and then we can draw samples from $p_0 \approx q_0$ by gradually denoising $\boldsymbol {x}_t$ with $\boldsymbol {\epsilon }_\theta (\boldsymbol {x}_t,t)$ to predict noise. Because of the iterative nature of these models, the computational cost is often higher than other DGMs (e.g. GANs) when scaling to higher resolutions or more complex data types. Many efforts have been devoted to improving the sample efficiency, including the training-free ODE solvers [10] and the distillation methods [11] with some extra training.

**Figure 1.**
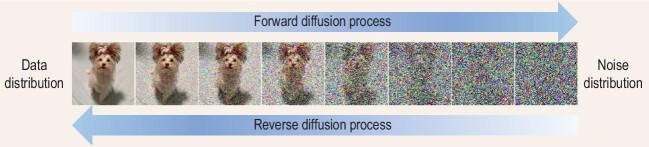
An illustration of diffusion models (adapted from [5]).

In many generation tasks, e.g. text-to-image generation [7], we would have some input (e.g. text prompts). One key technique for such applications is classifier-free guidance [12], which trains two weight-sharing models $\boldsymbol {\epsilon }_\theta (\boldsymbol {x}_t,t,y)$ and $\boldsymbol {\epsilon }_\theta (\boldsymbol {x}_t,t,\varnothing )$ for the noise prediction models, where *y* denotes the input (e.g. text prompt) and $\boldsymbol {\epsilon }_\theta (\boldsymbol {x}_t,t,y)$ is the conditional model. Here, we use $\varnothing$ as a special ‘empty’ token for the unconditional model. In a practical implementation, Ho and Salimans [12] chose to randomly set *y* to the unconditional identifier $\varnothing$ with some pre-specified probability. Classifier-free guidance then combines these two models as $\hat{\boldsymbol {\epsilon }}_\theta (\boldsymbol {x}_t,t,y) := (1+s)\boldsymbol {\epsilon }_\theta (\boldsymbol {x}_t,t,y) - s \boldsymbol {\epsilon }_\theta (\boldsymbol {x}_t,t,\varnothing )$ to trade off the text-image alignment and the sample diversity. The hyperparameter *s* is known as a ‘guidance scale’, where a larger *s* usually improves the text-image alignment, but reduces the sample diversity. By choosing a proper guidance scale, pre-trained large-scale text-to-image diffusion models can generate images with comparable quality to human artists.

Besides images, diffusion models (often with proper extension of the guidance) have been adopted for generating high-quality data across various domains, including speech, three-dimensional (3D) contents, human motions, videos and molecules. Specifically, diffusion models can imitate the voice of speaking or singing for a specific person and the generated voices are sometimes hard to distinguish [13]; diffusion models can lift the dimension from two dimensions to three dimensions and generate high-fidelity 3D contents without any 3D training data [14]; diffusion models have also been employed to synthesize human motion corresponding to a given text description [15]; by training with a large amount of video data, diffusion models can even generate short-term videos that are editable by different text prompts with representative systems such as Sora [16] and Vidu [17]. Besides, in bioinformatics and computational biology, diffusion models can facilitate the computational design of proteins and small molecules [18], potentially beneficial to drug discovery and molecular interaction modeling.

For future work, there are various challenges to be addressed in order to apply diffusion models to generate complex data. First, the dependency on large-scale training data to learn a reliable denoising function is one concern, especially in domains where such data are scarce (e.g. 3D contents) or sensitive (e.g. medical images). A possible way forward is to combine the generation of diffusion models with exploration by reinforcement learning methods to effectively interact with the real world. Additionally, the integration of domain knowledge or employing semi-supervised learning approaches could alleviate the dependency on extensive training data. Second, the current design for diffusion models mainly focuses on continuous domains, such as images, videos or audio, but it is hard to train diffusion models for discrete data, such as text. The performance of state-of-the-art diffusion models in language modeling is still worse than autoregressive models. As diffusion models have the potential for parallel decoding for multiple tokens, it is potentially valuable to study how to apply diffusion models in text distributions. Furthermore, the fusion of diffusion models with other generative frameworks like GANs or variational auto-encoders could spawn novel hybrid models with improved generation capabilities. Also, exploring the applicability of diffusion models in emerging domains like augmented reality, virtual reality and real-time multimedia synthesis presents exciting avenues. Lastly, the development of interpretable and controllable diffusion models could foster a deeper understanding and better control over the generated data, which is crucial for critical applications like healthcare and autonomous systems. Through concerted efforts in addressing these challenges and exploring new directions, the evolution of diffusion models is poised to significantly impact the landscape of data generation and even beyond, such as building adversarially robust classifiers from a pre-trained diffusion model [19] or adopting diffusion models to model human behaviors as the policy distribution [20] in reinforcement learning.
